# Improved Fuzzy Logic System to Evaluate Milk Electrical Conductivity Signals from On-Line Sensors to Monitor Dairy Goat Mastitis

**DOI:** 10.3390/s16071079

**Published:** 2016-07-13

**Authors:** Mauro Zaninelli, Francesco Maria Tangorra, Annamaria Costa, Luciana Rossi, Vittorio Dell’Orto, Giovanni Savoini

**Affiliations:** 1 Università Telematica San Raffaele Roma, Via di Val Cannuta 247, 00166 Rome, Italy; 2 Department of Health, Animal Science and Food Safety (VESPA), Università degli Studi di Milano, Via Celoria 10, 20133 Milan, Italy; francesco.tangorra@unimi.it (F.M.T.); annamaria.costa@unimi.it (A.C.); luciana.rossi@unimi.it (L.R.); vittorio.dellorto@unimi.it (V.D.); giovanni.savoini@unimi.it (G.S.)

**Keywords:** fuzzy logic, electrical conductivity, mastitis, dairy goats

## Abstract

The aim of this study was to develop and test a new fuzzy logic model for monitoring the udder health status (HS) of goats. The model evaluated, as input variables, the milk electrical conductivity (EC) signal, acquired on-line for each gland by a dedicated sensor, the bandwidth length and the frequency and amplitude of the first main peak of the Fourier frequency spectrum of the recorded milk EC signal. Two foremilk gland samples were collected from eight Saanen goats for six months at morning milking (lactation stages (LS): 0–60 Days In Milking (DIM); 61–120 DIM; 121–180 DIM), for a total of 5592 samples. Bacteriological analyses and somatic cell counts (SCC) were used to define the HS of the glands. With negative bacteriological analyses and SCC < 1,000,000 cells/mL, glands were classified as healthy. When bacteriological analyses were positive or showed a SCC > 1,000,000 cells/mL, glands were classified as not healthy (NH). For each EC signal, an estimated EC value was calculated and a relative deviation was obtained. Furthermore, the Fourier frequency spectrum was evaluated and bandwidth length, frequency and amplitude of the first main peak were identified. Before using these indexes as input variables of the fuzzy logic model a linear mixed-effects model was developed to evaluate the acquired data considering the HS, LS and LS × HS as explanatory variables. Results showed that performance of a fuzzy logic model, in the monitoring of mammary gland HS, could be improved by the use of EC indexes derived from the Fourier frequency spectra of gland milk EC signals recorded by on-line EC sensors.

## 1. Introduction

In lactating dairy goats, mastitis is one of the most important factor that can affect animals’ health status. It can be due by an intramammary infection (IMI), caused by a pathogenic microorganism, and it may be responsible for important economic losses because it can reduce the yield and quality of milk. Early detection, during milking, can help farmers improve animals’ health management and quantity/quality of milk produced. Many indexes have been studied in order to achieve this goal. Within those, the electrical conductivity (EC) of milk has been widely evaluated for this purpose [1,2,3,4,5,6,7,8].

The EC of milk is usually measured in milliSiemens per cm (mS/cm) and it indicates the ability of the solution to conduct an electric current between an anode and a cathode with a surface of 1 cm^2^ at a distance of 1 cm. The anions and the cations present in the milk are the species responsible for this ability [7]. They are mainly: Na^+^, K^+^, and Cl^−^ [8,9,10]. The concentration of these ions depends by different ionic flows that follow both cellular and paracellular pathways [11]. When an IMI is present, the milk shows higher concentrations of Na^+^ and Cl^−^ [12]. As a consequence, the milk EC measurement results increase [13].

Nevertheless, for dairy goats, good performance in detecting udder health status (HS) by the use of milk EC has not been possible. Published studies on this topic are scare and although some authors have demonstrated that milk EC signals can increase in infected goats [10,14,15], when major pathogens are the cause of the infection [16], algorithms and multivariate models that are able to achieve detection performance during milkings, and equal to those already reached for dairy cows, are still not reported [17].

For example, our research group, studying a univariate model based on evaluations of time series of gland milk EC data has found a specificity of 65% and a sensitivity of 81% [18]. This low performance is in line with results of other authors [10,19] which suggest that in order to reach a better accuracy it is necessary to: (1) consider the intrinsic variation of animals (as in cows); (2) avoid the use of simple thresholds; and (3) also use other significant factors in the model.

In line with these suggestions, our research group has studied a multivariate model based on daily milk EC and yield, and developed using fuzzy logic technology [20]. This technology allows one to develop multivariate models that translate the general knowledge of a specific field into a formal mathematical model suitable for computer processing [21]. In order to build a fuzzy logic system, three steps have to be performed: “*fuzzification*”, “*fuzzy inference*” and “*defuzzification*” [22]. The *fuzzification* phase consists in the transformation of each input variable of the model into a “linguistic variable”, where values are terms rather than numbers. A membership function, with an output range from zero to one and a triangular or trapezoidal shape, is defined for each term. The membership function describes, for each real value of the input variable, its grade of membership to the fuzzy term. In the *fuzzy inference* phase, the available knowledge of the modeled system is used. A set of rules, based on the “IF … THEN” structure, and the linguistic variables identified during the *fuzzification* phase, is formalized. The outputs obtained from the application of these rules are transformed back into real values in the final phase, called *defuzzification*. This phase is performed through specific areas, defined through the membership functions of the output variable, which translate the output of the *fuzzy inference* in a single numerical value (generally in the range between 0 and 1) using different calculation techniques (such as the center of gravity).

In dairy cows, this technology has been adopted with interesting results. In a study aimed to control lameness and mastitis in cows, some authors [23] achieved mastitis detection specificities that ranged between 88.3% and 77.4% using a fuzzy logic model and different definitions of mastitis. In another study, carried out to develop a fuzzy logic method to classify mastitis status in cows milked with an automatic milking system, other authors [24] reported sensitivities that ranged from 83.9% to 92.9% and specificities between 75.8% and 93.9%, and always on the basis of different definitions of mastitis. Finally, in a research conducted by another group of authors [25] with the target to reduce the number of FP cases produced by a previously developed detection system [26], a fuzzy logic model that achieved a sensitivity of 100% and a specificity >99.5% is described. In dairy goats, similar results have not been reached. In our experience, the use of fuzzy logic technology to evaluate milk EC data, in comparison with other univariate or multivariate models built with mathematical or statistical approaches, offer the advantages of being easy to interpret, modify and adapt [20]. The translation of the general knowledge into membership functions and rules, applied to the selected linguistic variables, was quite easy and different setups were considered, in order to obtain the better model accuracy, without any significant problems. Nevertheless, the performance reached was a specificity of 69% and a sensitivity of 81%. Even though better than other models [18,20], this accuracy still cannot be considered high enough if compared with results obtained in dairy cows [27]. New indexes, able to better characterize the milk EC signal in the case of not healthy (NH) glands, have to be found and used in order to improve the accuracy obtained.

In agreement with this target, our research group has applied spectral analysis to milk EC signals. This analysis allows one to characterize a signal, as well as its pattern, through its spectrum. The spectrum is calculated from the signal by specific mathematical operators, such as the Fourier Transform (FT), the Discrete Fourier Transform (DFT) or the Fast Fourier Transform (FFT), in the case algorithms, optimized and suitable for computer elaboration, are used. As a result of the spectral analysis performed, new qualitative and quantitative indexes were identified, namely the bandwidth length and the three main frequency peaks (FFT P_n_, n = 1–3) of the Fourier frequency spectrum of the milk EC signal [28,29]. These indexes showed significant mean values in the case of NH glands. In details, the mean value of the bandwidth length increased [29], lower means of frequencies were observed for all of the three main peaks [28], and higher means of amplitudes were found when all of the three main peaks were considered [28]. These results describe how the EC signal pattern changed in the time domain in the case of NH glands: it was generally characterized by slower fluctuations (due to the lower frequencies of the three main peaks) and by a more irregular trend (due to the higher amplitudes of the three main peaks and the increased bandwidth length). These results are in accordance with a previous study carried out on dairy cows [30]. Investigating the relationship between udder HS and different indexes based on the milk EC, the authors have reported that the statistical variance of all valid EC measures (σ^2^_EC_) increased from healthy to infected quarters with a greater difference in the case of clinical infected quarters. Nevertheless, in this study the variations of the milk EC signal were evaluated through a general index as the statistical variance. The indexes identified in our studies [28,29] allowed us to better characterize the EC signals, in the case of NH glands, from a qualitative and quantitative point of view. For this reason, they should be useful to improve the accuracy of a multivariate model that discriminates the gland HS of dairy goats.

The aim of this study was to develop and test a new multivariate model, using the fuzzy logic technology, for the monitoring of mammary gland HS of dairy goats. The model considered, as input variables, the milk EC signal, acquired on-line for each gland by dedicated sensors, the bandwidth length and the frequency and the amplitude of the first main peak of the Fourier frequency spectra of the recorded milk EC signals.

## 2. Materials and Methods

### 2.1. Animals and Farm Management

Eight second-parity Saanen goats, at the beginning of lactation, were randomly selected for the trial from a herd of 400 animals. Goats were fed twice a day with a common lactating basal diet for the entire experimental period on the basis of their nutritional requirements [31]. Goats were milked twice a day at 7:00 a.m. and 5:00 p.m. A low line side-by-side milking parlor with 16 milking units for each side was used. A system vacuum of 40 kPa, a pulsation rate of 90 cycles/min, and a pulsation ratio of 60:40 were set.

### 2.2. Experimental Design, Milk Sample Collection and Analyses

The experiment was carried out for six month. During each morning milking, milk samples were collected after the teat disinfection (with chlorhexidine-moistened towels) and the discharging of the first milk streams. From each mammary gland of the animals’ trial group, two individual milk samples were taken for each day of milking and lactation stage (LS) evaluated (0–60 Days In Milking (DIM); 61–120 DIM; 121–180 DIM).

A total amount of 5592 milk samples were collected during the trial. From these samples, 2796 were used for bacteriological analysis (i.e., one for each gland and day of milking in the LS considered) according to the International Dairy Federation standard method [32] while the other 2796 were analyzed for somatic cell counts (SCC) and following the International Dairy Federation recommendations [33].

Milk samples were classified according to the results of microbiological tests and SCC [10,14,16,17,18,20,28,29]. In detail, milk samples were considered as from NH glands when:

(1) bacteriological analyses were positive for IMI;
(2) bacteriological analyses were negative for IMI and SCC were more than 1,000,000 cells/mL on two or more consecutive sampling days for non-physiological causes. In this context, an increase of SCC was considered due to physiological causes when: (a) it occurred in both glands in an isolated analysis that was followed by SCC < 1,000,000 cells/mL in a subsequent analysis; and (b) the end of lactation was been reached.

The remaining milk samples were classify as from healthy glands. When milk samples were collected, milk EC signals were also measured and stored by the data acquisition system. Milk EC signals acquired were from each mammary gland of the animals’ trial group.

### 2.3. Milk Electrical Conductivity Measures and Data Acquisition System

**Figure 1:**
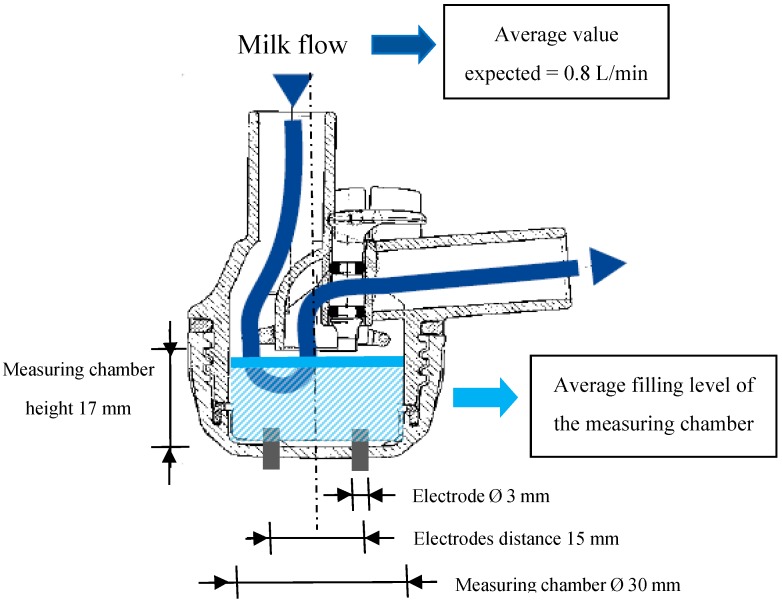
Dimensions of the EC sensor head placed at the base of each individual teatcup.

In order to measure the milk EC from each gland, four experimental milking clusters were used. These clusters were developed in previous experiments [15,18,20,28,29], modifying commercial milking units (Vanguard, Interpuls S.P.A., Albinea, RE, Italy). Each milking cluster included two EC sensors. Each EC sensor was made by a couple of stainless cylindrical electrodes (Figure 1) placed at the base of each individual teatcup. This hardware allowed the measuring of the specific EC of milk (in milliSiemens (mS/cm)) while it was flowing from the gland to the milk line. Furthermore, a flow detector was placed inside each short milk tube of the milking cluster. It was made of an additional couple of cylindrical stainless electrodes that, measuring a signal proportional to the filling level of the short milk tube, allowed us to monitor the beginning and the end of each milking and to avoid or correct possible data errors due to the presence of milk residues in the milking claws.

The hardware components used to acquire the EC signals were also the same as in previous studies [15,18,20,28,29]. In details, four analog conductivity boards (output range 0–10 V, accuracy ± 0.1%), placed in a separate room next to the milking parlor and connected to an analog/digital conversion board installed in a personal computer (DAQCard AI-16E-4, National Instruments, Austin, TX, USA—with a resolution of 12 bit and a total sampling rate of 250 kS/s), were used to measure the electrical signals from the milking clusters. Furthermore, through a customized software application developed using LabVIEW 8.02 (National Instruments), acquired data were sampled with a rate of 1 Hz and stored as txt files using the goat identity farm number, date and time to name each file. Other technical details on the milking groups, on the sensors and a complete block schema of the whole recording system are provided in [28].

In previous studies [28,29], these sensors were shown to measure the EC of milk with mean values higher than those reported in other experiments. This fact did not affect the results obtained and it was explained supposing an average quantity of milk, in the measurement chamber of the EC sensors, not equal to the value expected by the calibration procedure. However, in order to improve the accuracy of the milk EC measurements, a new round of laboratory tests was carried out before the start of the experiment. The effects of different milk flow rates on the measurements made by the EC sensors were checked. A solution of water and chlorine-based detergent for the milking machine was used as test fluid. The detergent was added to the water to increase its EC up to 6 mS/cm. Two EC sensors (included in the same milking cluster) were tested at constant liquid flow rates—from 0.4 L/min to 1.2 L/min in incremental steps of 0.2 L/min—using a suitable artificial udder equipped with a flow regulator. Ten repetitions were made for each flow rate investigated, for a total number of 100 readings (i.e., 10 repetitions per five flow rates per two sensors). For each repetition performed, approximately 5 L of test fluid passed through the milking cluster and the electrical signals measured from the sensors were stored by the recording system. As following steps: (1) the mean value of the electrical signal acquired during each reading was calculated; (2) for each flow rate tested, the mean value of all the ten repetitions performed was calculated; (3) considering the differences between the mean value measured at 0.8 L/min and the mean values measured at the other flow rates tested, the measurement accuracy of the sensor was estimated as the mean value of the errors found. The flow rate of 0.8 L/min was taken as reference because it was considered as the average milking flow rate expected in the following field tests.

Similar laboratory tests were also performed in order to check the linearity of the EC sensors and calibrate them. The same kind of fluid test was used, but in this case, detergent was added to the water to increase its EC from 4 mS/cm up to 12 mS/cm, by incremental steps of 2 mS/cm. All of the EC sensors were tested at a constant liquid flow rate of 0.8 L/min. Ten repetitions for each EC level and for each experimental milking cluster were made for a total of 400 readings (i.e., 10 repetitions per five EC levels per eight sensors). Also in these cases, for each repetition performed, approximately 5 L of test fluid passed through the milking cluster and the electrical signals measured from the sensors were stored by the recording system. As following steps: (1) the electrical mean value of each reading was calculated; (2) the electrical mean value for each EC level tested was calculated; (3) on the resulting data, a linear regression was performed for each sensor tested. At the end of these tests, obtained results allowed the setup of each EC sensor. During the field tests, no other calibration procedures were performed on the EC sensors used.

### 2.4. Elaborations of the Acquired EC Signals

**Figure 2:**
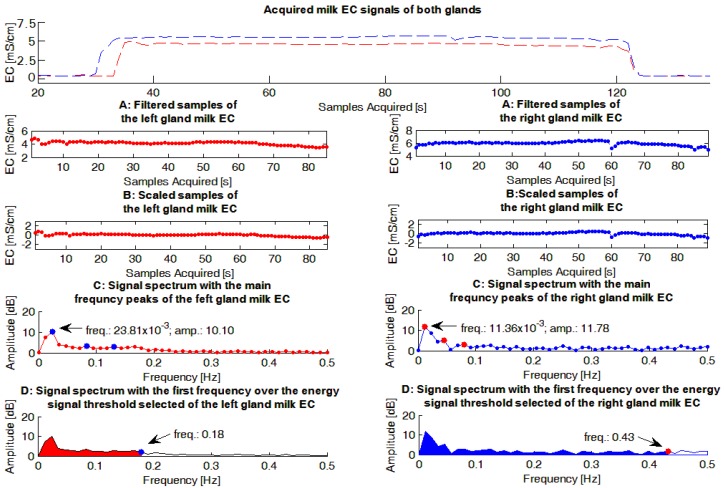
Example of gauges obtained from the milk electrical conductivity (EC) signals acquired within a milking. Furthermore, the following graphs report: (**A**) the sequences without the signal samples related to the start and the end of milking; (**B**) the sequences where the mean value of each sequence have been subtracted to each signal sample acquired; (**C**) the Fourier frequency spectra of the previous sequences of signal samples and the three main frequency peaks and (**D**) the bandwidth length of the signal (also colored to be easily highlighted).

The milk EC signals were evaluated by a dedicated Matlab routine (The Mathworks, Natick, MA, USA). The main steps performed by the software routine (Figure 2), for each EC signal evaluated, were:

(1) Using data from the flow detector included in each short milk tube, sensor samples related to the start and the end of a milking were deleted from the sequence in order to build the vector $S_{n}$.
(2) Through the Matlab function *mean*, the average value of $S_{n}$ was calculated and subtracted to each sample of the sequence to build the vector: $S_{n}^{'}$ according to the following formula:
  $$S_{n}^{'}=\;S_{n}-\;\bar{S_{n}}$$
  This step was performed in order to have a Fourier frequency spectrum of each EC signal with a null peak at the frequency of zero, and consequently, a scaled graph in the frequency domain useful in identifying the most important peaks of the spectrum.
(3) Using the Matlab function *fft*, the Fourier frequency spectrum was calculated ($Sp_{f}$) applying the Fast Fourier Transform (FFT) operator. The parameters of the function were the vector $S_{n}^{'}$ and its dimension (i.e., the *length*($S_{n}^{'}$)).
(4) The positive part of the spectrum was obtained ($Sp_{f}^{'}$) considering the values of the vector $Sp_{f}$ in the range of frequencies between 0 and Fs/2 (where Fs was the EC signal sampling rate, i.e., 1 Hz) according to the following formula:
  $$Sp_{f}^{'}=\;Sp_{f}\;\text{with}\;0<f<\;\frac{Fs}{2}$$
(5) The total energy of the EC signal was evaluated ($E_{Tot}$), translating in Matlab the following formula:
  $$E_{Tot}=\frac{Fs}{k-1}\;\overset{k}{\underset{i=1}{\sum }}{\left|Sp_{f}^{'}\left[i\right]\right|}^{2}$$
  where: $Sp_{f}^{'}$ was the positive part of the spectrum, k was the number of samples of the positive part of the spectrum (i.e., the dimension of the vector $Sp_{f}^{'}$ equal to the value *length*($Sp_{f}^{'}$)) and Fs was the sampling rate of the EC signal (i.e., 1 Hz).
(6) threshold of 95% ($T_{95}$) of the EC total signal energy ($E_{Tot}$) was selected and the first frequency that surpassed that level was considered as the last frequency of the signal bandwidth (Figure 2). The threshold of 95% of the total signal energy was chosen because it was considered a reasonable value able to highlight the most important information conveyed by each spectrum, after evaluating the average signal/noise ratio provided by the experimental spectra obtained. To perform this step:
  (a) according to the following formula:
    $$E_{Tot,\;f}=\frac{Fs}{k-1}\;\overset{f}{\underset{i=1}{\sum }}{\left|Sp_{f}^{'}\left[i\right]\right|}^{2}$$
    a vector of incremental total energies was calculated, using the Matlab function *cumsum* instead of the simple function *sum*;
  (b) according to the following condition:
    $$E_{Tot,\;f_{0}}>T_{95}\times E_{Tot}$$
    the first frequency ($f_{0}$) that surpassed the total signal energy threshold was calculated, using the Matlab function *find* applied to the vector $E_{Tot,f}$.
(7) The three highest frequency peaks of each spectrum (FFT_P_1,2,3_), and for each of them the corresponding frequency and amplitude, were calculated. To complete this step:
  (a) a combination of the Matlab functions *diff* and *find* was used as follows:
    $$Sp_{f}^{'}\_local\_max\;=\;find(diff(diff(Sp_{f}^{'})\;>\;0)\;<\;0)$$
  (b) the first three values of the vector $Sp_{f}^{'}\_local\_max\;$were selected and considered as the most representative peaks (FFT_P_1,2,3_) of the spectrum evaluated.
(8) A figure that summarized the main steps performed by the Matlab routine, and the results obtained, was built and stored. An example of these figures is shown in Figure 2.

Before investigating the data acquired during the trial, another elaboration of the EC signals was performed. For every morning milking and mammary gland, a new EC variable $\left(EC^{'}\right)$ was defined as the deviation of the EC of the day $(EC_{t}$) from a predicted value $\left(EC_{t}^{'}\right)$ calculated through the following *moving-average* model:

$$EC_{t}^{'}=\;\frac{1}{N}\;\overset{N}{\underset{K=1}{\sum }}EC_{t-k}N=10$$

According to the findings of other authors [34], ten previous days of milking were considered in the model (N = 10).

### 2.5. Fuzzy Logic and Model Setup

In this study, the input variables used for the *fuzzification* phase were: “Maximum EC” (i.e., the maximum value of milk EC shown within mammary glands on the same day of milking), “Deviation EC” (i.e., the relative deviations of milk EC between measured and estimated values), “Bandwidth Length” (i.e., the first frequency that surpassed the threshold of 95% of the total milk EC signal energy, acquired for each mammary gland and day of milking), “Peak Frequency” and “Peach Amplitude” (i.e., the frequency and amplitude of the first highest frequency peak of each Fourier frequency spectrum—FFT_P_1_—calculated for each milk EC signal acquired for each mammary gland and day of milking). The membership functions of the input variables were mainly derived from the scientific literature [10,16,20,24,25,28,29,35]. Details on the terms and shapes used are given in Table 1.

The determination of the udder HS of goats with membership functions equal to *low*, *middle*, *high*, and *very high* probability of NH glands were the outcomes of the combined input variables. All of the rules, applied to the input variables: *maximum EC*, *deviation EC*, *bandwidth length*, *peak frequency* and *peak amplitude*, are reported in Table 2.

**Figure 3:**
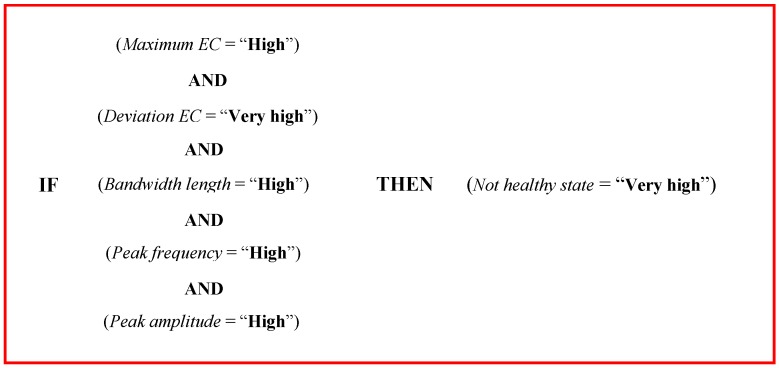
Example of a rule used in the *fuzzy inference*.

An example of a rule, used in the *fuzzy inference* phase and derived from data reported in Table 2, is reported in Figure 3.

The meaning of the rule reported in Figure 3 is that when *maximum EC* was high, *deviation EC* was very high, *bandwidth length* was high, *peak frequency* and *peak amplitude* were high, the fuzzy logic model classified the probability to have a case of a NH gland as very high. Using the same logic structure reported in Figure 3 and data reported in Table 2, all the 324 rules used in the *fuzzy inference* can be described.

Through the center of gravity of the area below a specific geometric shape, output values of the *fuzzy inference* phase were transformed back into a single number. This geometric shape was obtained from the superimposition of the membership functions of the output variable and it was dynamically modified by the output values of the *fuzzy inference*. The x-axis of the calculated center of gravity was set as the *defuzzified* result of the fuzzy system and used in the statistical analyses. All of these computational evaluations were carried out using the Fuzzy Logic Toolbox of Matlab.

### 2.6. Statistical Analyses

Statistical analyses conducted in this study were performed using R (R Core Team, Vienna, Austria—version 3.2.3, 2015) as the statistical software tool. Relationships between mammary gland HS and SCC, EC, bandwidth length, frequency and amplitude of the peak FTT_P_1_ were studied in order to validate the data acquired. Values of EC and SCC were log transformed (base 10) to normalize their distributions. The Shapiro-Wilk test was used to confirm the normal distribution of all the variables under study.

Associations between the explanatory variables and SCC, EC, bandwidth length, frequency and amplitude of the peak FTT_P_1_ were evaluated using a linear mixed-effects model (procedure *lme* [36] of the package *nlme* “*Linear and Nonlinear Mixed Effects Models*”, version 3.1-122). The linear model fitted was the following:

$$Y_{ijkl}=\mu +\;HS_{i}+\;LS_{j}+\;HS\times LS_{ij}+\;g_{k\left(l\right)}+a_{l}+e_{ijkl}$$

where Y was the SCC or EC, bandwidth length, frequency and amplitude of the peak FTT_P_1_; μ was the mean; HS*_i_* was the effect of health status (*i* = 0–1; 0 = healthy; 1 = NH); LS*_j_* was the effect of lactation stage (*j* = 1–3; 1 = 0–60 DIM; 2 = 61–120 DIM; 3 = 121–180 DIM); HS × LS*_ij_* was the interaction between health status and lactation stage; g*_k(l)_* was the random effect of the gland (*k* = 1–2; 1 = left, 2 = right) nested in the goat (l = 1–8) [37]; a*_l_* was the random effect of the goat (l = 1–8) and e*_ijkl_* was the residual error. Furthermore, an autoregressive covariance structure (correlation = corAR1 [38]) was used to account for the repeated measurements in the same goat [37,39].

The accuracy reached by the fuzzy logic model was evaluated through comparisons between the output values of the *defuzzification* phase and a set of specific cut-off levels. These levels were chosen considering the range of values of the output variable of the fuzzy logic model. This range was always from 0 to 1. Therefore, a set of cut-off levels that included values from 0.1 to 0.9, with incremental steps of 0.1, was considered as a reasonable segmentation of this range able to show the performances of the fuzzy logic model under study. When the *defuzzified* value exceeded a specific cut-off level, an alarm was reported. Otherwise, the predicted status was considered as healthy. For each mammary gland and day of milking, after the first ten observations, a comparison between the alarms and the observed status was performed and classified as: true positive (TP), if an alarm was reported by the model and the corresponding milk sample was classified as from NH gland; false negative (FN), if no alarm was reported and the corresponding milk sample was classified as from a NH gland. Furthermore, when milk samples were classified as from healthy glands, each result was considered true negative (TN), if no alarm was reported or false positive (FP), if an alarm was reported by the fuzzy logic model.

On the basis of all the comparisons performed, sensitivities and specificities achieved by the fuzzy logic model were calculated. In this context, the sensitivity represents the percentage of glands correctly identified as NH with respect to all of the cases of milk samples classified as belonging to NH glands:

Sensitivity = True Positive/(True Positive + False Negative) × 100

The specificity indicates the percentage of glands correctly identified as healthy in respect to all of the cases of milk samples classified as belonging to healthy glands:

Specificity = True Negative/(False Positive + True Negative) × 100

For each cut-off level considered, a pair of sensitivity and specificity was calculated. In order to define the accuracy reached by the fuzzy logic model, a specific pair of sensitivity and specificity was selected among those calculated. Generally, the final cut-off level selected for a diagnostic test depends on the needs and/or on the gold standards, when available, of the specific application. In dairy cows, an average sensitivity of 80% has been reported [40] as the gold standard of human observation, although it may be affected by variables such as the skills of the milker and the severity of the case. In line with this result, the final cut-off level that was selected in this study was the value that allowed reaching a sensitivity of at least 80%. As a consequence, a pair of sensitivity and specificity was identified, among those calculated, and considered as the level of accuracy reached by the fuzzy logic model under study.

## 3. Results

The overall EC sensor accuracy in laboratory tests carried out at different flow rates, was 1.46% with no relevant differences between the two EC sensors evaluated. Within the range of the EC levels investigated, linear trends were confirmed for all the EC sensors tested. In Table 3, the parameters of the linear regressions performed are reported. These parameters were used for the recording system setup before the field tests were carried out.

Eleven milk samples, within those collected, were classified after microbiological evaluation as contaminated because more than three different bacteriological species were found (probably because for those samples a wrong sampling procedure had been carried out). The prevalence of positive samples was 49.3% (N = 1378, Table 4) with Coagulase-negative *Staphylococcus* as the most prevalent mastitis agent (41.1%, Table 4). Eight glands, from five different goats, were infected from 91 to 120 days of milking (Table 5). The prevalence of glands with SCC > 1,000,000 and without pathogenic microorganisms was 1.9% (N = 53, Table 6) and no cases of SCC > 1,000,000 due to physiological causes were observed. The overall prevalence of samples from NH glands was 51.2% (Table 6) and no cases of clinical mastitis were observed.

Mean values of SCC were significant higher in NH glands than in healthy ones (5.46 ± 0.03 (logSCC) vs. 5.35 ± 0.02, Table 7) and showed significantly increased levels during the progress of lactation (5.30 ± 0.02 (logSCC), 5.47 ± 0.02 and 5.55 ± 0.02, Table 7). The interaction between the HS and LS was not significant.

Not healthy glands showed significantly higher values of milk EC (9.07 ± 0.06 (mS/cm) vs. 7.64 ± 0.07, Table 8). Furthermore, a significantly lower mean value of milk EC was observed in the first stage when compared with the other lactation stages (7.01 ± 0.06 (mS/cm) vs. 9.32 ± 0.07 and 10.22 ± 0.01). The interaction between the HS and LS was not significant also for these cases.

Regarding the spectra evaluated, data showed a normal distribution for the relative parameters investigated: bandwidth length, frequency and amplitude of the peak FFT_P_1_. The bandwidth length showed a significantly higher mean value in NH glands (0.26 ± 0.004 (Hz) vs. 0.23 ± 0.003; Table 9) and significantly lower levels between different lactation stages (0.25 ± 0.0004 (Hz), 0.24 ± 0.004 and 0.22 ± 0.006). Mean values of FFT_P_1_ frequency were significantly lower in NH glands (11.63 ± 0.32 × 10^−3^ (Hz) vs. 13.66 ± 0.42 × 10^−3^, Table 10) and significantly lower in different lactation stages (13.72 ± 0.38 × 10^−3^ (Hz), 12.30 ± 0.46 × 10^−3^ and 9.49 ± 0.29 × 10^−3^). The peak’s mean amplitude was significantly higher in NH glands (37.19 ± 0.6 (dB) vs. 30.68 ± 0.69, Table 11) and during the progress of lactation (26.87 ± 0.96 (dB), 39.09 ± 0.76 and 40.43 ± 1.28). However, the interaction between the HS and LS was always not significant for all the mean values of the relative parameters investigated: bandwidth length, frequency and amplitude of the peak FFT_P_1_.

Lastly, the HS detection accuracy reached by the fuzzy logic model was investigated. Different cut-off levels were evaluated and for each of them, a pair of sensitivity and specificity was calculated. Results showed that the best accuracy was: specificity equal to 78% and sensitivity of 80%, with a cut-off level equal to 0.6 (Table 12). The cut-off level was determined in order to reach a sensitivity of at least 80%. Consequently, the resulting pair of sensitivity and specificity was defined as the accuracy reached by the multivariate model studied.

## 4. Discussion

The range of flow rates investigated during laboratory tests showed that the design of the EC sensor allowed one to isolate a stable quantity of milk and, as a consequence, to achieve a fine measurement accuracy. This sensor was developed in order to measure the EC signal of milk, on-line and from each gland, without affecting the flow of milk, the vacuum of the milking system and to be a device usable in the most existing milking parlors. Having these targets in mind, a low number of components were added to a commercial milking cluster and no specific element was added to control the air that could enter through the claw or the teatcups. Nevertheless, the position of the couple of electrodes at the base of the teatcup, the presence of a flow sensor in the short milk tube and the sampling rate used to acquire the EC signals limited possible reading mistakes and allowed us to achieve a fine measurement accuracy in the range of flow rates tested in the laboratory. Additionally, the relationships between the EC levels and the measurements of the EC sensors showed a good linearity and allowed us to perform a calibration of each EC sensor through a specific angular coefficient. These angular coefficients were lower than those found in a previous experiment carried out by our research group [28]. This result was probably due to the different flow rate used in laboratory tests, performed at different EC levels (0.8 vs. 0.6), and allowed us to measure, in the following field tests, a specific EC of milk with a better accuracy.

The percentage of positive samples of the whole study, found after microbiological evaluation of milk, was high (49.3%). However, the most prevalent mastitis agent was coagulase-negative *Staphylococcus* that has the potential to become a chronic infection [41] and consequently to affect the prevalence of positive samples in a study that involves a small number of animals, managed separately from the others animals of the farm.

In milk samples from NH glands, mean values of SCC and EC were significantly higher. Similar results are reported also by other authors [10,14,16] who observed a significant increase of SCC and EC in case of infected glands. During the progress of lactation, mean values of SCC and EC showed to increase significantly between the first, the second, and the third lactation stages. Also these results agree with previous studies in which higher levels of SCC and EC were measured with the progress of lactation [10,16]. Finally, when a gland was infected, the milk EC values were higher than the moving average of the ten days before in the 68% of the cases that where continuously classified as NH.

Nevertheless in this study, EC mean values measured during milkings were higher than those reported by other authors under similar conditions (i.e., HS and stage of lactation) [8,10,14,16,17,19] and lower than those found by our research group in some previous studies [20,28]. Although the small group of animals used in this experiment could explain these results, we think that an additional factor could be that the average quantity of milk in the measurement chamber of the EC sensors was not equal to the value assumed by the calibration procedure. This volume is affected by the average flow rate used in the setup procedure. Therefore, obtained results suggest that: (1) this flow rate was more correct than the value used in previous studies; (2) it is still not adequate for the average milk flow rate that can be found in a real milking parlor; and (3) the calibration procedure is crucial, but it has to be performed simulating, as much as possible, the real operative conditions even though this could be difficult with an experimental milking system and an artificial udder. Further investigations will be useful to achieve the best measurement accuracy for these EC sensors. In any case, all these aspects did not affect the performance of the fuzzy logic model studied. The input variables used in the model were EC indexes based on relative EC values or elaborations of EC data that involved, for each sample of the corresponding sequence, the subtraction of the mean value of the milk EC signal recorded.

Before evaluating the accuracy achieved by the fuzzy logic model investigated, relationships between EC indexes used as input variables, glands HS, and lactation stages were studied. The results showed that mean values of bandwidth lengths were significantly higher in the case of NH glands and lower between the first and the second lactation stage, if compared to the mean value found in the third lactation stage. Also mean values of frequency and amplitude of the peak FFT_P_1_ showed significant results. Lower values of frequencies were found in the case of NH glands and in different lactation stages. Higher values of amplitudes were measured in NH glands and during the progress of lactation. These results were in line with other studies carried out by our research group [28,29]. Furthermore, they confirm how the milk EC signal pattern changes in case of NH glands and during the progress of lactation. In all of these cases, the signal pattern is generally characterized by slower fluctuations (due to the lower frequencies of the first main peak) and by a more irregular trend (due to the higher amplitudes of the first considered peak). Since a better characterization of milk EC signals could be useful to improve the accuracy of multivariate models that monitor the gland HS of dairy goats, these results confirm that these EC indexes may be a way to reach this goal.

In order to achieve a sensitivity of at least 80%, the cut-off level selected for the studied fuzzy logic model was 0.6. With this cut-off level, the resulting specificity and sensitivity of the model were 78% and 80%, respectively. These results were better than those obtained by other univariate and multivariate models that use the EC of milk to detect mammary gland HS of dairy goats. Diaz et al. [10], analyzing data from three different farms and using a simple threshold based on the index LEC (base 10 logarithm of the milk EC measurement), reported specificities that ranged between 22% and 47% and sensitivities between 60% and 93% (with a threshold of 5.20 mS/cm and depending on the farm considered). In detail, considering different cut-off levels in order to reach sensitivity of at least 80%, specificities found were 40% with a cut-off of 5.0 mS/cm in farm 1; 27% with a cut-off of 5.1 mS/cm in farm 2; and 35% with a cut-off of 5.4 mS/cm in farm 3. Romero et al. [14], studying the effect of the milking fraction on the milk EC measurements, reported that the best performance obtainable was with a defined threshold of 5.20 mS/cm a specificity of 50% and a sensitivity of 70%. In a subsequent study, Romero et al. [17] also investigated the ability to detect gland HS of different algorithms based on the daily measurements of milk EC. In this study, data from 18 goats were collected for a month. After the first two weeks, animals were exposed to various unfavorable health situations for the mammary gland that might increase the mastitis probability. After IMI establishment, animals were milked over the following two weeks and 19 algorithms were evaluated. These algorithms were based on the EC and the EC ratio of collateral glands of the same goat (RAT_EC_ = maximum EC/minimum EC), and used data recorded before the day of the infection establishment to predict a range of normality (through an Autoregressive Integrated Moving Average model). The best results obtained in this study were: (a) for the index EC, a specificity of 75% and a sensitivity of 58.3% considering four days before establishment of infection as data size and the “*rule 1*” (i.e., deviations exceeding three standard deviations) as the cut-off level to identify a “positive case”; (b) for the index RAT_EC_, a specificity of 88.9% and a sensitivity of 44.4% considering 4–8 days before establishment of infection as the data size and the “*rule 1*” or “*rule 3*” (deviations exceeding 5% of the moving average value) as the cut-off level to identify a “positive case”. Additionally, our research group investigated a similar univariate model [18]. Considering daily measurements of milk EC and 10 previous values to calculate, through a *moving-average* model, relative deviations of EC between measured and estimated values, in this study we found a specificity of 65% and a sensitivity of 81%, setting as threshold of EC deviations a cut-off level of 7%. Finally, a multivariate model based on fuzzy logic technology was also tested by our research group in a previous study [20]. As input variables of the model, relative deviations of milk EC and milk yield were considered. The accuracy achieved by the model was a specificity of 69% and a sensitivity of 81%, setting a cut-off level of 0.7.

Although this study has to be considered as a pilot study, its results suggest that the EC indexes used as input variables of the fuzzy logic model could allow one to achieve a better accuracy in the detection of dairy goats’ health status. Furthermore, as suggested by other authors [10,19], results reached in this study confirm that to develop useful monitoring systems: (1) simple thresholds shared among animals have to be avoided; (2) animals’ intrinsic variability has to be considered using relative values instead of absolute measurements; and (3) a better characterization of milk EC signals in the case of NH glands could be a way to improve the accuracy of these types of algorithms.

Nevertheless, the accuracy obtained by the fuzzy logic model evaluated still cannot be considered high enough if compared with results obtained in dairy cows. Kramer et al. [23], in a study on the control of lameness and mastitis in cows, evaluated the accuracy of a fuzzy logic model. Setting the block-sensitivity to be at least 70%, the authors found in mastitis detection specificities that ranged between 88.3% and 77.4%, depending on the different definitions of mastitis (e.g., udder treatments, or udder treatments and/or SCC over 400,000/mL). In this study, block-sensitivity was calculated, considering as true-positive, the “mastitis blocks” (i.e., uninterrupted sequences of “days of mastitis”) were one or more alerts were been given in the first five days. Cavero et al. [24], in a study on cows milked with an automatic milking system, reported for a fuzzy logic method developed to classify mastitis status specificities from 75.8% to 93.9% and sensitivities that ranged from 83.9% to 92.9%, depending on the different definitions of mastitis (e.g., udder treatments performed in the case of SCC > 100,000 cells/mL or SCC > 400,000 cells/mL). De Mol and Woldt [26], in a study designed to reduce the number of FP cases produced by a previously developed detection model [27], tested a fuzzy logic method and reported a specificity of 99.5% and a sensitivity of 100%. All clinical cases were correctly classified, and the resulting number of FP alerts from a subset of 25 cows that did not show any sign of mastitis was reduced from 1266 to 64 by applying the fuzzy logic method studied.

A possible way to achieve a better accuracy in the monitoring of dairy goats HS could be the use of the stage of lactation as an input variable of the fuzzy logic model. Electrical conductivity indexes used in the present study also showed significant trends during the progress of lactation. Therefore, the use of this parameter could allow a better characterization of milk EC signals and consequently to permit the development of monitoring systems with better performances. Nevertheless, in order to use these algorithms in a real milking parlor a “Radio Frequency IDentification” (RFID) system able to identify each animal, and consequently to record each lactation stage, would be necessary. This could limit the applicability of the monitoring system developed. For this reason, this parameter was not included in the fuzzy logic model tested.

As a more general result, this study confirms that fuzzy logic technology is a valid way to develop multivariate models for the monitoring of dairy goats’ health status. The translation of basic knowledge, provided by scientific literature and by our previous experiments, into membership functions and rules applied to the selected linguistic variables was easy and when different membership functions, in terms of shapes and outputs, and different rules were considered in order to obtain the better setup of the model studied, no significant problems were found. Therefore, through a better knowledge of the relationship between the milk EC signal and the mammary gland HS, we think that this technology would be suitable for the development of monitoring systems able to reach positive results, in terms of herd management, and also for the goat farming agricultural sector.

## 5. Conclusions

In the monitoring of udder health status of dairy goats, the present study showed that a fuzzy logic model could be improved by the use of EC indexes derived from Fourier frequency spectra of milk EC signals recorded by on-line EC sensors. When bandwidth lengths, frequencies and amplitudes of the first main peaks were considered as input variables of the model, better results in detecting mammary gland heath status were reached than those reported for other multivariate models proposed in the scientific literature.

## Figures and Tables

**Table 1:** Membership functions for the milk electrical conductivity (EC) traits considered in the study. These traits were: (1) Maximum EC; (2) Deviation EC; (3) Bandwidth Length; (4) Peak Frequency and (5) Peak Amplitude.

Trait	Function	Shape	Point of Characterization
Maximum EC	Low	Trapezoidal	(0; 1) (3.5; 1) (7; 0)
	Middle	Triangular	(3.5; 0) (7; 1) (10.5; 0)
	High	Trapezoidal	(7; 0) (10.5; 1) (14; 1)
Deviation in EC	Low	Trapezoidal	(0; 1) (0.04; 1) (0.11; 0)
	Middle	Triangular	(0.04; 0) (0.11; 1) (0.18; 0)
	High	Triangular	(0.11; 0) (0.18; 1) (0.25; 0)
	Very high	Trapezoidal	(0.18; 0) (0.25; 1) (0.7; 1)
Bandwidth Length	Low	Trapezoidal	(0; 1) (0.09; 1) (0.13; 0)
	Middle	Triangular	(0.13; 0) (0.17; 1) (0.21; 0)
	High	Trapezoidal	(0.21; 0) (0.25; 1) (1; 1)
Peak Frequency	Low	Trapezoidal	(0; 1) (0.009; 1) (0.013; 0)
	Middle	Triangular	(0.009; 0) (0.013; 1) (0.017; 0)
	High	Trapezoidal	(0.013; 0) (0.017; 1) (0.025; 1)
Peak Amplitude	Low	Trapezoidal	(0; 1) (15; 1) (30; 0)
	Middle	Triangular	(15; 0) (30; 1) (45; 0)
	High	Trapezoidal	(30; 0) (45; 1) (60; 1)

EC, electrical conductivity.

**Table 2:** Rules of the *fuzzy inference* phase about the electrical conductivity (EC) traits considered in the study. The rules are applied to the input variables: *maximum EC*, *deviation EC*, *bandwidth length*, *peak frequency* and *peak amplitude* and, with membership functions equal to *low*, *middle*, *high*, and *very high*, they provide, as outcomes, the probability to find a not healthy gland.

Fuzzy Inference Rules						Bandwidth Length								
						Low			Middle			High		
						Maximum EC			Maximum EC			Maximum EC		
						Low	Middle	High	Low	Middle	High	Low	Middle	High
Peak Amplitude	Low	Peak Frequency	Low	Deviation EC	Low	None	None	None	None	None	None	None	None	None
					Middle	None	None	None	None	None	None	None	None	None
					High	None	None	None	None	None	None	None	None	Low
					Very high	None	None	None	None	None	Low	None	Low	Middle
			Middle	Deviation EC	Low	None	None	None	None	None	None	None	None	None
					Middle	None	None	None	None	None	None	None	None	Low
					High	None	None	None	None	None	Low	None	Low	High
					Very high	None	None	Low	None	Low	Middle	Low	Middle	Very high
			High	Deviation EC	Low	None	None	None	None	None	None	None	None	Low
					Middle	None	None	None	None	None	Low	None	Low	High
					High	None	None	Low	None	Low	High	Low	High	Very high
					Very high	None	Low	Middle	Low	Middle	Very high	Middle	Very high	Very high
	Middle	Peak Frequency	Low	Deviation EC	Low	None	None	None	None	None	None	None	None	None
					Middle	None	None	None	None	None	None	None	None	Low
					High	None	None	None	None	None	Low	None	Low	High
					Very high	None	None	Low	None	Low	Middle	Low	Middle	Very high
			Middle	Deviation EC	Low	None	None	None	None	None	None	None	None	Low
					Middle	None	None	None	None	None	Low	None	Low	High
					High	None	None	Low	None	Low	High	Low	High	Very high
					Very high	None	Low	Middle	Low	Middle	Very high	Middle	Very high	Very high
			High	Deviation EC	Low	None	None	None	None	None	Low	None	Low	High
					Middle	None	None	Low	None	Low	High	Low	High	Very high
					High	None	Low	High	Low	High	Very high	Middle	Very high	Very high
					Very high	Low	Middle	Very high	Middle	Very high	Very high	Very high	Very high	Very high
	High	Peak Frequency	Low	Deviation EC	Low	None	None	None	None	None	None	None	None	Low
					Middle	None	None	None	None	None	Low	None	Low	High
					High	None	None	Low	None	Low	High	Low	High	Very high
					Very high	None	Low	Middle	Low	Middle	Very high	Middle	Very high	Very high
			Middle	Deviation EC	Low	None	None	None	None	None	Low	None	Low	High
					Middle	None	None	Low	None	Low	High	Low	High	Very high
					High	None	Low	High	Low	High	Very high	Middle	Very high	Very high
					Very high	Low	Middle	Very high	Middle	Very high	Very high	Very high	Very high	Very high
			High	Deviation EC	Low	None	None	Low	None	Low	High	Low	High	Very high
					Middle	None	Low	High	Low	High	Very high	Middle	Very high	Very high
					High	Low	High	Very high	Middle	Very high	Very high	Very high	Very high	Very high
					Very high	Middle	Very high	Very high	Very high	Very high	Very high	Very high	Very high	Very high

EC, electrical conductivity.

**Table 3:** Parameters of the linear regressions performed on the electrical conductivity (EC) sensors used in the experiment and evaluated at different EC levels, from 4 mS/cm up to 12 mS/cm, by incremental steps of 2 mS/cm.

Sensor	Angular Coefficient	R^2^
*1*	1.88	0.98
*2*	1.72	0.99
*3*	1.73	0.98
*4*	1.80	0.98
*5*	1.81	0.97
*6*	1.76	0.98
*7*	1.66	0.97
*8*	1.72	0.97

**Table 4:** Distribution of pathogenic microorganisms found in infected mammary glands.

Isolated Bacterial Strains	N	%
Coagulase-negative *Staphylococcus (CNS)*	1148	41.1
*Escherichia coli*	36	1.3
*Streptococcus spp.*	136	4.9
*Pseudomonas spp.*	47	1.7
Contaminated	11	0.3
*BC negative*	1418	50.7

**Table 5:** Distribution of infected cases along mammary glands, different goats and ranges of days of milking.

Glands	Goats	Days of Milking
2	1	30–60
6	4	60–90
8	5	90–120

**Table 6:** Distribution of samples for each mammary gland health status considered.

Health Status of Glands	N	%	Samples with Positive Bacteriological Analyses and SCC < 1,000,000 (cells/mL)		Samples with Positive Bacteriological Analyses and SCC > 1,000,000 (cells/mL)		Samples with Negative Bacteriological Analyses and SCC > 1,000,000 (cells/mL)	
			N	%	N	%	N	%
Not healthy	1431	51.2	1155	41.3	223	8.0	53	1.9
Healthy	1365	48.8	--	--	--	--	--	--

**Table 7:** Overall means and standard errors (S.E.) of SCC (log) of gland milk samples according to mammary gland health status and lactation stages.

Health Status of Glands	Days in Milking			
	*0–60* Mean ± S.E. (logSCC)	*61–120* Mean ± S.E. (logSCC)	*121–180* Mean ± S.E. (logSCC)	*0–180* Mean ± S.E. (logSCC)
Not healthy	5.34 ± 0.03	5.49 ± 0.02	5.58 ± 0.03	5.46 ^A^ ± 0.03
Healthy	5.28 ± 0.03	5.43 ± 0.03	5.48 ± 0.02	5.35 ^B^ ± 0.02
All	5.30 ^x^ ± 0.02	5.47 ^y^ ± 0.02	5.55 ^z^ ± 0.02	5.40 ± 0.02

^A,B^ means in the same column with different uppercase superscripts differ significantly (*p* < 0.01); ^x,y,z^ means in the same row with different uppercase superscripts differ significantly (*p* < 0.05).

**Table 8:** Overall means and standard errors (S.E.) of electrical conductivity (mS/cm) of gland milk samples according to mammary gland health status and lactation stages.

Health Status of Glands	Days in Milking			
	*0–60* Mean ± S.E. (mS/cm)	*61–120* Mean ± S.E. (mS/cm)	*121–180* Mean ± S.E. (mS/cm)	*0–180* Mean ± S.E. (mS/cm)
Not healthy	7.56 ± 0.09	9.57 ± 0.08	10.25 ± 0.11	9.07 ^A^ ± 0.06
Healthy	6.55 ± 0.07	8.88 ± 0.13	10.07 ± 0.23	7.64 ^B^ ± 0.07
All	7.01 ^X^ ± 0.06	9.32 ^Y^ ± 0.07	10.22 ^Y^ ± 0.1	8.48 ± 0.07

^A,B^ means in the same column with different uppercase superscripts differ significantly (*p* < 0.01); ^X,Y^ means in the same row with different uppercase superscripts differ significantly (*p* < 0.01).

**Table 9:** Overall means and standard errors (S.E.) of the bandwidth length, according to mammary gland health status and lactation stages.

Health Status of Glands	Days in Milking			
	*0–60* Mean ± S.E. (Hz)	*61–120* Mean ± S.E. (Hz)	*121–180* Mean ± S.E. (Hz)	*0–180* Mean ± S.E. (Hz)
Not healthy	0.26 ± 0.005	0.26 ± 0.007	0.25 ± 0.008	0.26 ^a^ ± 0.004
Healthy	0.24 ± 0.006	0.23 ± 0.005	0.22 ± 0.007^3^	0.23 ^b^ ± 0.003
All	0.25 ^X^ ± 0.004	0.24 ^x^ ± 0.004	0.22 ^Y,y^ ± 0.006	0.24 ± 0.004

^a,b^ means in the same column, with different uppercase superscripts differ significantly (*p* < 0.05); ^X,Y^ means in the same, row with different uppercase superscripts differ significantly (*p* < 0.01); ^x,y^ means in the some row, with different lowercase superscripts differ significantly (*p* < 0.05).

**Table 10:** Overall means and standard errors (S.E.) of the frequency of the first most representative spectrum peak (FFT_P_1_), according to mammary gland health status and lactation stages.

Health Status of Glands	Days in Milking			
	*0–60* Mean ± S.E. (Hz)	*61–120* Mean ± S.E. (Hz)	*121–180* Mean ± S.E. (Hz)	*0–180* Mean ± S.E. (Hz)
Not healthy	12.83 ± 0.31 × 10^−3^	11.89 ± 0.65 × 10^−3^	9.30 ± 0.29 × 10^−3^	11.63 ^a^ ± 0.32 × 10^−3^
Healthy	14.44 ± 0.64 × 10^−3^	13.03 ± 0.55 × 10^−3^	10.39 ± 0.91 × 10^−3^	13.66 ^b^ ± 0.42 × 10^−3^
All	13.72 ^X^ ± 0.38 × 10^−3^	12.30 ^Y^ ± 0.46 × 10^−3^	9.49 ^Z^ ± 0.29 × 10^−3^	12.47 ± 0.51 × 10^−3^

^a,b^ means in the same column, with different uppercase superscripts differ significantly (*p* < 0.05); ^X,Y,Z^ means in the same, row with different uppercase superscripts differ significantly (*p* < 0.01).

**Table 11:** Overall means and standard errors (S.E.) of the amplitude of the first most representative spectrum peak (FFT_P_1_), according to mammary gland health status and lactation stages.

Health Status of Glands	Days in Milking			
	*0–60* Mean ± S.E. (dB)	*61–120* Mean ± S.E. (dB)	*121–180* Mean ± S.E. (dB)	*0–180* Mean ± S.E. (dB)
Not healthy	28.95 ± 0.67	40.71 ± 0.96	40.68 ± 1.38	37.19 ^a^ ± 0.60
Healthy	25.07 ± 0.69	36.27 ± 1.24	39.23 ± 3.39	30.68 ^b^ ± 0.69
All	26.87 ^X^ ± 0.96	39.09 ^Y^ ± 0.76	40.43 ^Y^ ± 1.28	32.97 ± 0.59

^a,b^ means in the same column with different lowercase superscripts differ significantly (*p* < 0.05); ^X,Y^ means in the same, row with different uppercase superscripts differ significantly (*p* < 0.01).

**Table 12:** Accuracy reached by the fuzzy logic model in terms of sensitivity and specificity at different cut-off levels.

Cut-Off Level	Sensitivity (%)	Specificity (%)
0.90	56	92
0.80	69	86
0.70	76	83
0.60	80	78
0.50	85	74
0.40	90	68
0.30	93	59
0.20	97	47
0.10	99	27
